# Dilemmas and Repercussions of Workplace Violence against Emergency Nurses: A Qualitative Study

**DOI:** 10.3390/ijerph19052661

**Published:** 2022-02-25

**Authors:** Mei-Chi Hsu, Mei-Hsien Chou, Wen-Chen Ouyang

**Affiliations:** 1 Department of Nursing, I-Shou University, Kaohsiung City 82445, Taiwan; hsu6889@gmail.com (M.-C.H.); mei.hsien@msa.hinet.net (M.-H.C.); 2 Department of Geriatric Psychiatry, Jianan Psychiatric Center, Ministry of Health and Welfare, Tainan City 71742, Taiwan; 3 Department of Nursing, Shu-Zen Junior College of Medicine and Management, Kaohsiung City 82144, Taiwan; 4 Department of Psychiatry, College of Medicine, Kaohsiung Medical University, Kaohsiung City 80708, Taiwan

**Keywords:** workplace violence, patient and visitor violence, nurse, emergency department, dilemma, antecedent, repercussion

## Abstract

Nurses received the highest rate of workplace violence due to their close interaction with clients and the nature of their work. There have been relatively few qualitative studies focus on nurses’ perceptions of and experiences with the antecedents, dilemma and repercussions of the patient and visitor violence (PVV), leaving a considerable evidence gap. The aim of this study was to explore nurses’ experience of PVV in emergency department, the impact of PVV on quality of care, and supports needed after exposure to such incidents. We conducted semi-structured interviews with a purposive and snowball sample of nurses, and analyzed the content of the interview transcripts. A total of 10 nurses were approached and agreed to participate. Those participants ranged in age from 24 to 41 years old, eight female and two male nurses, and the majority of them (80%) held a university Bachelor degree in nursing. The average time in nursing practice was 7.2 years. We conceptualized five analytical themes, which comprised: (1) multifaceted triggers and causes of PVV; (2) experiences following PVV; (3) tangled up in thoughts and struggle with the professional role; (4) self-reflexivity and adjustment; and, (5) needs of organizational efforts and support following PVV. This paper provides compelling reasons to look beyond solely evaluating the existence of workplace, and considering the perceived professional inefficacy, impacts of being threatened or assaulted in nurses. There are also urgent needs in provision of prevention and management of workplace training programs to ensure the high-quality nursing care.

## 1. Introduction

Workplace violence (WPV) is prevalent in healthcare and remains a complex and serious occupational hazard in the health care settings [1,2]. The incidents of WPV in emergency departments (EDs) are increasing and have become one of the most significant and problematic issues [3]. The intrinsic work feature of EDs is different from other health care settings [4]. For example, WPV in EDs is most likely to occur as a result of unmanageable conflict that escalates and turns into an impulsive and unplanned physical or non-physical aggression. Overt dominance and power and control over others may be less among the perpetrators. This results in violence in EDs, which is different from other forms of violence.

Research dating back to the late 1990s has provided the evidence linking EDs and WPV [5,6] that 42–57% of the ED workers were physically assaulted over the 1-year study period, and 72% of them over the span of their careers. The overall prevalence (71%) of WPV was high [7]. Thus, a zero tolerance policy for WPV has been suggested which identifies actions and behaviors that will not be tolerated within the EDs [8,9]. Although the broad-based review of the published literatures has highlighted the important findings and limitations relating to the prevalence of WPV in EDs, the definitions of WPV varied. Individual studies often adopt differing definitions of WPV. For example, threats of physical assault are defined or classified in different studies, as either physical violence or verbal abuse [3]. 

Recently, the COVID-19 outbreak has brought unique challenges to the EDs [10]. As EDs are at the frontline against this pandemic in managing both COVID-19 and non-COVID patients, the pandemic has necessitated a rapid overhaul of ED operations, necessary training and staffing in preparation for a sustained response. To minimize the infection rate among the healthcare teams in Taiwan, all hospitals have responded promptly and strived through comprehensive and multidisciplinary approaches. However, incidents of WPV in EDs increased during the pandemic [11]. In their recent study, McGuire et al. [11] have twice surveyed multidisciplinary ED staff, and found that WPV incidents were apparently increased: 2.53 incidents per 1000 visits during pandemic in comparison with 1.13 incidents per 1000 visits at 3 prior months, and 1.24 incidents per 1000 patient visits in the previous year. Thus, there is a positive association between WPV incidents and the COVID-19 case rate. WPV can happen anywhere at any time, even more often during the COVID-19 pandemic [11].

The high prevalence of WPV among nurses is not a new phenomenon. Nurses are particularly at high risk from WPV including physical aggression, verbal abuse, threats, and harassment because of the nature of their work, frequent and longer contacts with patients or families, provision of direct care or insufficient staffing within their work environments [4]. A review of 136 articles has reported that prevalence of violence against nurses ranged from 24.7% to 88.9% [12]. A current large study [13] observed that the prevalence of WPV among physicians and nurses was 47.9% (1264/2637) in the past year. Particularly among nurses, the prevalence of physical and psychological violence was 11.7% and 58.5%, respectively. 

In Taiwan, ED/intensive care unit (ICU) were at high risk for most occupational incidents than those in other wards [2,14]. Approximately 30% of ED/ICU nurses reported physical violence compared to 20% of ward nurses [14] and 5.4% in the outpatient department [2].

Generally, WPV can be classified into four basic types: Type I criminal intent, Type II customer/client, Type III worker-on-worker, and Type IV personal relationship [15,16,17]. Type I means that the perpetrator has no relationship with the targeted organization or employees, but often committing a crime in conjunction with the violence. Type II customer/client violence includes patients and their families or visitors (patient and visitor violence, PVV). Type III is that perpetrator is a current or past employee of the workplace. The factor is often interpersonal or work-related conflicts. Type IV means that perpetrator has a personal relationship with the victim (the nurse) outside of work, but does not have a relationship with the workplace. Although there is scant systematic evidence to reveal the true prevalence of type II incidents, the literatures have suggested that PVV is the most common source of violence against health care workers [4,7,12,18]. It has been reported that patients accounted for nearly 90% of this violent behavior. For physical violence, 64.3% is performed by patients, and one-third (30.2%) by their family/visitors [12]. 

EDs are a highly dynamic and stressful care environment that affect staff and patients’ outcomes, with particular risks for staff’s well-being and quality of care. There are numerous adverse work factors identified, such as heavy work demands under time pressure, high patient turnover, staff shortages, increased patient morbidities, exposure to violent individuals, and lack of effective workplace violence prevention programs and protective policy or regulations [7]. These factors contribute to high stressful situations for patients, families, and staffs. These persistent stressful situations increase the risk for PVV ubiquitous [7,19,20]. Studies conducted in EDs have suggested that long wait times, excess noise, overcrowding, feeling a lack of caring, being given bad news related to diagnosis or prognosis, and ineffective interpersonal relationships are all possible risk factors for WPV [7,21,22,23]. 

The issue of WPV in the health care settings is further compounded by the under-reporting of incidents to hospital. This raises a question on the underlying reasons why staffs choose not to report the incidence of WPV. Several studies have provided possible reasons for the under-reporting of violence. They may be due to conflicts between one’s ethical principles, and one’s role of obligations and duty of care; staffs may view the incident as an indication of their own inability to manage; staffs do not want to attract any attention; feel guilt, shame; or fear of negative consequences, and being blamed [8,18,24,25,26]. Nurses’ responses to WPV are complex; they include a broad spectrum of negative physical, psychological, emotional, functional, and work-related outcomes [2,3]. Violence experience is generally associated with adverse health outcomes, such as an increased risk of type 2 diabetes [27] and physically injured in an assault [12]. 

Nurses’ attitude towards WPV is a complex concept. It involves modulating one’s state or behavior in a given situation. It has been assumed that if underlying attitudes are changed, more enduring changes in behavior could be expected by the changing attitudes. A person may subsequently produce many specific changes in overt behavior. Therefore, attitude issues are important in exploratory studies related to WPV [28,29,30].

Compared to other professionals, nurses particularly are at the highest rate of WPV due to the nature of their tasks and responsibilities. Most research on WPV in health care has been retrospective, explanatory, exploratory, and descriptive in nature, particularly in relation to its prevalence, incidence rates, and impacts on staff in a variety of clinical settings. Davey, et al., [31] have carried out a qualitative study explored how violence exposure is widespread and issues surrounding violence against healthcare providers. Others have examined the causes of violence against nurses exercised by patients and/or their relatives e.g., [32], and nurses’ perceptions of and experiences of WPV perpetrated by patients, their relatives, colleagues and superiors e.g., [33]. 

To date, there is still a lack of studies on the detailed antecedents and consequences of violent events and nurses’ exposure to PVV. Based on current available literatures, there are insufficient data regarding the antecedents and outcomes of PVV experiences in ED nurses allowing us to explore further. To capture when PVV is occurring and find effective strategies to manage it, the full extent, related factors, and magnitude of the problem must first be evaluated. A qualitative approach extends understanding by illustrating how the impact of PVV on nurses may go beyond workload, as the pattern of PVV are shaped by features specific to the work environment, wider context and particular situations.

Considering the significant impacts of PVV on nurses, it is crucially important to explore the experiences of PVV. The aim of this explorative qualitative study was to explore nurses’ experience of PVV in emergency department, the impact of PVV on quality of care, and supports needed after exposure to such incidents. This study focused on understanding nurses’ perceptions of and experiences with the antecedents, dilemma and consequences of PVV in ED.

## 2. Materials and Methods

### 2.1. Design

A qualitative descriptive study using semi-structured interview, was conducted in 2020. This study was designed to facilitate an in-depth exploration of the comprehensive perspective of nurse’s experience as a victim of PVV and related workforce issues. This research has also extended the approach to illustrate: (1) how the impact of PVV on ED nurses which may go beyond physical injuries; and (2) how a psychological trauma may be detrimental to the quality of patient care and nurses’ wellbeing. 

### 2.2. Nurse Recruitment and Consent

The researchers developed a recruitment plan of estimating the eligible and accessible participants. A combination of purposive and snowball sampling was selected. Inclusion criteria were registered nurses who work full-time and provide direct patient care in the ED, having being assaulted by patients, their families and/or visitors, and agreeing to provide informed consent. Nurses who did not provide direct care in the ED, and were not eligible to participate in the study were excluded in the study. In addition, nurses who took part in the present study, were asked to provide referrals to recruit more nurses required for this study. Therefore, trust and rapport are especially important in snowball sampling for a participant to agree to identify other potential participant or their group. Once in contact with a potential participant, the researchers further explained the study.

A total of ten nurses were approached and agreed to participate. Those participants ranged in age from 24 to 41 years old, and the majority of them (80%) held a university Bachelor degree in nursing. The average time in nursing practice was 7.2 years.

### 2.3. Interview Guide

The interview guides were developed. The guides covered a wide range of topics. They were kept purposefully broad and open-ended to allow participants to intensively describe their experiences. The interview included questions about PVV related experiences, impact and repercussions, and dilemmas, needs, and requests for the future.

Prior to study started, a pilot interview was conducted to ensure clarity in meaning. After the pilot interview, only minor adjustments of question contents were made. The interview began with general questions about the nature of the job and broader context around the definitions of PVV, before focusing on their experiences as being a victim. All interviews were conducted in a private office in the workplace.

### 2.4. Data Collection

The researchers approached the potential participants after they indicated their volunteer agreement to participate in this study. As part of the informed consent process, all participants received a project information sheet, which outlines the study purpose, interview recording and transcribing procedures, participant rights and privacy issues. Semi-structured interviews were conducted in an open framework based on the interview guides. The individual interviews lasted 1–2 h each. With consent, we audiotaped and transcribed the interviews. The focus of the interview was to gather rich and meaningful data capturing how the phenomenon (PVV) was experienced, the nature of PVV, and an essence that held this phenomenon and the lived experiences together [34]. The interview guides were kept purposefully broad and open-ended to allow participants to deeply describe their experiences. Field notes were taken during the interviews to maximize rigor. These experiences were then discussed with the research team. These steps offered the possibility of exploring the experiences of PVV and its dynamics, in the context in which it occurred [34]. Data saturation occurs at the point at which findings no longer reveal new information within further interviews, while carrying out analysis.

### 2.5. Researcher Reflexivity

Three researchers with professional backgrounds in mental health, public health, nursing, and medicine as well as having expertise in violence-related research conducted the interviews. The researchers carefully discussed preconceptions and experiences in the field before commencing the study. Since, the researchers had no prior experiences with the ED working conditions and no prior collaboration with the workplace, they were more detached, and less personal during the interviews. Their professional backgrounds and career in mental health also allowed them to possess a special sensitivity, empathy, and understanding of the violent issues. Thus, the reflexivity [35] from both sides might be a strength of the study, which facilitated the interpretation of interview data and subsequent analysis. 

### 2.6. Rigor

The concept of rigor in qualitative research, such as transferability, confirmability, credibility and dependability created by Lincoln and Guba [36] was adopted in the present study. Strategies for ensuring rigor encompassed such as thick description, provision of rich detail of the participants’ impressive experiences, saturation, frequent debriefing sessions and meetings, and the researchers’ professional background. Contents of interviews were transcribed and compared for accuracy. Clear descriptions of the study design and appropriate quotations of participants’ own words were also provided to facilitate transferability and dependability. 

### 2.7. Data Analysis

Qualitative data were analyzed using content analysis [37]. The researchers discussed data saturation, and the findings showed that the data saturation was reached. The researchers checked the transcripts against the original recording for accuracy. A constant comparative process between two researchers was conducted; this facilitated to refine the identified themes and ensure rigor and quality to the present study [38]. The transcripts were repeatedly read and analyzed, and open coding was independently, iteratively, and thematically conducted by the researchers. A series of codes was developed. Once meaning was developed from these codes they were then grouped into themes [39]. Findings were also triangulated with literature available and participants’ experiences. These procedures served to maintain objectivity and avoid potential bias in the analysis of qualitative data. 

### 2.8. Ethical Considerations

The study protocol for this trial was approved by the Institutional Review Board (IRB) of the participating hospital (registered No. EMRP70108N). As the present study was of a sensitive and potentially distressing nature, participants were informed about the purpose of the study. Their participation in the present study was completely anonymous, voluntary, and confidential.

## 3. Results

In this paper, five analytical themes regarding the experiences of nurses emerged: multifaceted triggers and causes of PVV, experiences following PVV, tangled up in thoughts and struggle with the professional role, self-reflexivity and adjustment, and needs of organizational efforts and support following PVV (Table 1).

After analysis of participants’ data, we identified the recurring theme of ‘multifaceted triggers and causes of PVV’; the essence of this theme was extracted from the following excerpt:

### 3.1. Multifaceted Triggers and Causes of PVV

#### 3.1.1. Unpredictability and Complexity of Diverse Clinical Conditions

Nurses in the EDs provided direct nursing care to patients with diverse critical conditions and in complex and unpredictable situations, which exposed them to stress and threats. These stress and uncontrollable conditions included providing care to mentally unstable patients, and interacting with their family members or visitors who might not be in good and stable mental conditions. Therefore, nurses are at great risk for threat and violence.

“*The young drunken man still balled his fists after arriving at the emergency department. … Things didn’t get any better when a doctor and I came in to stitch him up. The patient yelled at us and tried to get up while he was getting stitches. As I tried to prevent him from causing more damage to his wounds, he swung his arm and hit me,.. We were forced to call a police after this young drunk patient became more unstable, and combative*”.

(Nurse D)

“*That patient had multiple injuries and hence, needed quick treatments in order to prevent him from further getting more injuries or even death. The patient and his [the patient] families became quite anxious and stressful, and had conflicts with us. One of his families angrily accused us of something and then pushed me so hard that I fell down*”.

(Nurse F)

#### 3.1.2. Misestimation of Violent Situation

Nurses explained that they often ignored that they were in danger. They stated that they were unrealistic to believe that they could handle the violent situation and face it by themselves. Their knowledge and self-protection against PVV were insufficient.

“*I didn’t think so much at the moment, I just wanted to make him emotionally stable. I thought I could do it, but the patient attacked me with fists*”.

(Nurse A)

“*Everyone was busy. So, I thought I could deal with it [conflicts with the patient] by myself first. I really misestimated how dangerous the patient with mental disorder could become*”.

(Nurse D)

#### 3.1.3. Obstacles to Effective Teamwork

The teamwork was not sufficient and effective in violence management in the ED. Teamwork is the key. Nurses mentioned that the team’s function should be able to allow for quick recognition and proper action on violence.

“*Our team were having the meetings about the violent incident that we had. The cooperation of the whole team was actually not enough. We were not well-trained in proactive prevention and interventions of violence. We were really unaware that it is a part of our work in this emergency department*”.

(Nurse C)

“*I was injured while I was trying to restrain the patient. Unfortunately, our team was not well-coordinated. The restraint should not commence unless a full team, at least 5 people, was present at the scene. … We probably should notify the security guards first, not call them after something went worst*”.

(Nurse H)

### 3.2. Experiences Following PVV

#### 3.2.1. Invisible and Inevitable Physical and Psychological Trauma

Nurses consistently expressed that violence from patients/visitors was invisible and inevitable, which caused both physical and psychological trauma. However, most nurses still felt it was not worth reporting.

“*This is the second year I worked in the emergency department … It was not the first time that they [patients/visitors] yelled at me and other nurses. They attempted to humiliate us. Then they said that I was too sensitive and had no sense of humor*”.

(Nurse C)

“*We were taking care of an old lady… Her temper was really bad. She was so aggressive… really distressed. I attempted to calm her down. However, the more I talked to her, the more she wanted to hit and scream at me…*”.

(Nurse B)

“*I was shocked and sad. I didn’t expect that patient treated me like this. I was just trying to help him. This incident made me losing motivation for work*”.

(Nurse F)

#### 3.2.2. Impulse to Hit Back

Nurses stated that it was hard to be an empathetic person when they were attacked, and when the perpetrators were abusing them verbally using the offensive words. Nurses reported that they have zero tolerance to the perpetrators. Even though they understood very well that their ethical obligation was to maintain and respect an individual’s dignity, impulse control could be a challenge for them to have adequate self-control and convince themselves not to impulsively hit back. They jokingly expressed that their capacity of controlling impulse and behavior needed to be strengthened. However, the ethical dilemmas experienced by nurses is vivid.

“*At the time of being attacked by the perpetrator, I was so angry and felt so bad. I really wanted to resist and defend against him [the perpetrator]*”.

(Nurse A)

“*He shouted at me in front of a lot of people in the emergency department. I really wanted to scold him loudly and said to him “Are you crazy*”.

(Nurse I)

#### 3.2.3. Loss of Confidence

Nurses exposed to PVV also reported that they perceived themselves professional inefficacy, and confidence and enthusiasm diminished in caring of patients. This lived experience left nurses frustrated. It was a subject that nurses did not want to admit, and wonder if PVV might just be a part of their job in the ED because of the high frequency of violent events.

“*I am here to care for people. When I was attacked, I really felt that nurses must be so wrong, so they deserved to be scolded and beaten …, This made me wonder whether I should stay on this job or not*”.

(Nurse G)

“*Why should I be hurt so much by PVV here? It [PVV] only reminds me of leaving my job. Is there any dignity in this job*”?

(Nurse J)

### 3.3. Tangled Up in Thoughts and Struggle with the Professional Role 

#### 3.3.1. Encounters of Feeling Disrespected

The finding that nurses’ feeling of not being respected highlights a need of a healthy work environment to retain and support them. Nurses stressed that the issue of disrespect is one of their biggest concerns, particularly from the non-urgent patients who frequently used ED services. The feeling of disrespected can be illuminated by the following excerpts.

“*His [patient] symptoms, dyspeptic complaints and dizziness, were actually not that urgent. His families said that I was not professional and I was nobody. They treated me like I was a stupid nurse. … I felt powerless. This kind of behavior doesn’t help the nurse-patient relationship at all. Just the opposite, trust was broken, there’s going to be a rift in the nurse-patient relationship. … Feeling disrespected is a discouragement to me*”.

(Nurse E)

“*Their [patient/visitor] disrespect of nurses actually is having an impact on my working morale. I felt so bad when they treat nurses in a disrespectful way*”.

(Nurse G)

#### 3.3.2. Symptom Sequelae Following Violence 

Nurses experienced various conditions and injuries related to PVV. PVV caused symptom sequelae or injuries are not limited to bruises, black eyes, or large and painful contusions and cuts. Migraines, headaches, and hearing loss are also common.

“*My right eye and orbit were injured. I was very worried about my eye. I felt that my vision is losing gradually over time*”.

(Nurse D)

“*After I suffered from violence to the head, my hearing loss became severe. I am still suffering from such bad injury and physical and mental scars. Sadly, my hearing loss is a constant reminder of the violence*”.

(Nurse J)

#### 3.3.3. Fear of Retaliatory Acts of Violence and Future Violence at Work

The fears of retaliatory acts of violence and future violence at work are the long-term negative consequences for nurses experiencing PVV. The fear of being revenged in shadow can affect nurses’ lives and cause psychological distress. Thereby, the needs of receiving prompt help largely increased. The fear of being revenged might be more lasting and disturbing than physical injury. Furthermore, the fears might significantly help in understanding the links between PVV and the negative psychological and occupational consequences.

“*I didn’t dare to walk out alone for a period of time after experiencing violence by the brutal family member. … For a while after violence happened, whenever I walked outside, I fear of being attacked again and retaliated by that family. The memory of being intimidated always haunts me*”.

(Nurse A)

“*I was afraid of going to work. I was particularly concerned about being attacked again. No one can predict when or where it will happen. What I need most was someone who could tell me what to do, how to seek legal support, and give me advice*”.

(Nurse I)

### 3.4. Self-Reflexivity and Adjustment

In response to the violent incidences, nurses exhibit different attitudes. Nurses who had experienced the difficulty of rediscovery of themselves and their job role, stated that their adjustment was achieved through self-reflexivity. The process of self-reflexivity was deemed as a new learning experience. Some nurses have even stressed that PVV provided them with an opportunity to increase their confidence to re-think the importance of prevention and management of PVV in the ED. They expressed that such self-reflexivity and adjustment probably could mitigate the likelihood of being assaulted again and regain the confidence of nursing care. 

#### 3.4.1. Ineffective Communication Skills

Some nurses recognized that violent incidents were caused by poor communication skills. Difficulties in communication played a role in the occurrence of PVV.

“*I have to take part of the responsibility myself. It [violence] might be triggered by my facial expressions, tone of my voice and probably what I said to him [perpetrator]*”.

(Nurse F)

“*I* *felt I did not have the skill to deal with the situation. My de-escalating attempts were actually not working at all*”.

(Nurse H)

#### 3.4.2. Stress-Buffering Effects of Self-Affirmation

Some nurses commented on their positive experiences when affirming their own worthiness and value. They drew from these experiences to boost their ego when struggling with the consequences of PVV. They felt better under the self-affirmation condition. Their self-affirmation enhanced their belief as a professional nurse, reduced PVV’s harmful impact, and shaped their attitudes toward facing and caring the difficult and violent patients again.

“*All is well. I am strong. They [perpetrators] didn’t know what they were doing. … I had a patient apologize to me because he yelled at me. I knew he [patient] was confused, and he didn’t really want to hurt me…*”.

(Nurse H)

“*I was caring for her and later she was discharged after recovering from her discomfort. I got a lot of satisfaction from being able to help her. It’s really worthwhile and I want to do it again because it makes me feel like I’ve achieved something*”.

(Nurse I)

#### 3.4.3. Support from Management and Colleagues 

Nurses will seek support, encouragement, and empathic understanding from colleagues, friends, and family during the violent incidents and in the aftermath of incidents. The support from colleagues is crucial in providing the needed assistance to get through the impact of violence.

“*The first time I experienced the PVV, my leader cared about me, she came to help me immediately and later taught me how to complete the reporting process. Without her support I wouldn’t be OK. They helped me through*”.

(Nurse B)

“*My colleagues openly supported me when facing that patient’s threats, and I felt really supportive with them. It had a healing effect on me at the time*”.

(Nurse J)

“*My colleague switched that patient to another nurse for me because she knew my emotional burden of caring that patient was unbearable at the time*”.

(Nurse E)

### 3.5. Needs of Organizational Efforts and Support Following PVV

#### 3.5.1. Raising Awareness of Violence

Nurses expressed that it is important to take an active role in promoting changes such as facilitating practice change, raising awareness, and understanding guidelines, as well as considering how their response or behavior could impact on patient/visitor in busy emergent environments. Some nurses also pointed out the importance of nurses being well-prepared for the possibility of a threatening or violent situation. One important element was their capacity and awareness when facing warning signs. They need to increase sensitivity to violence, and be able to foresee possible precursors.

“*I have suggested that all nurses should improve our awareness and sensitivity in order to prepare ourselves on how to respond to the warning signs of workplace violence. Raising awareness of violence can prevent us from getting hurt*”.

(Nurse H)

#### 3.5.2. Guidelines and Policies

When facing PVV, nurses need managers to be concerned about the reinforcement of their capacity for violence prevention and management in addition to the adverse impact of events on them. The training program of violence is considered important as an approach of enhancing their knowledge of risk assessment of violence. Nurses place substantial value in the beneficial outcomes of interventions.

“*… it is necessary to hold continuing training and practical exercises, such as communication skills, restrains and risk assessment process for managing the risks of violence. The training should be practical, not theoretical …*”.

(Nurses C and E)

#### 3.5.3. Necessary Renovations of Security Equipment

Generally, the security equipment for prevention of violence is implemented at organizational level. Some nurses said that they felt much safer after the safety facilities in the ED were improved.

“*The hospital have changed or renovated some of the security equipment such as surveillance cameras and alarm systems in the aftermath of violent incidents*”.

(Nurse A)

“*We all need to know how to use the digital products if violence happens. For example, the digital recorders are placed in close proximity to nurses. Everyone should be familiar with the way of using it*”.

(Nurse H)

## 4. Discussion

This qualitative study provided a deeper understanding of nurses’ experiences and their perceptions of PVV. The study identified supportive needs of nurses following PVV from the viewpoint of nurses as victims. This study contributes to the literature by exploring specific antecedent and outcome variables related to PVV, which encompass health, wellbeing and occupational-related consequences, and self-efficacy beliefs. This study also highlighted the need for a more explicit recognition of the dilemma, antecedent factors and consequences in ED nurses. Our findings are consistent with the results from many previous studies that the impact of WPV and PVV in particular on nurses is associated with unpredictable psychological distress or trauma [40,41,42,43,44], difficult adjustment process [44], struggle with the role as a nurse [40,41,42,43,44], changes of the meaning of work [31,45], ineffective coping [46], decreased quality of care [31], and the needs of supports in the aftermath of incidents [40,41,42,43,44]. 

In the present study, we showed that antecedent factors included nurses’ perceptions of situational, environmental, occupational, personal, and organizational issues, and existing procedures of dealing with PVV as well as self-efficacy beliefs. We have also presented findings delineating nurses’ experiences and the focal antecedent factors and outcomes.

The cause of PVV is multifactorial. It has been shown that violence is perpetrated more frequently by patients than by visitors [18]. A current study indicated that caring for patients in EDs during their most vulnerable times was associated with increased risk of WPV [1]. In this study of PVV, we have also revealed the communication dilemma in the busy emergency department. This theme encompasses communication, care, support, trust, expectations. Having to discuss clinical management and related information with patients based on judgements of their clinical conditions was perceived as a challenge to nurses. Communication problems could be frustrating for both nurses and patients, and could lead to anxiety, increased stress, excessive demands and uncertainty in patients, which, consequently evoke PVV [18,24,47]. Education and training would include, for example, communication-skills training, and de-escalation skills.

The present study has also shown that PVV negatively affects nurses’ health outcomes and well-being, as evidenced by having symptoms such as anxiety, unpleased mood, and even trauma. This is consistent with the previous studies. WPV could result in psychological depletion of healthcare workers, encompassing impaired self-esteem [48], and poorer quality of life [49], which indirectly trigger negative emotions such as anxiety, emotional exhaustion, the presence of psychological distress, or even post-traumatic stress disorder symptoms [13,50], and development of sleeping disorders [14].

Our findings suggest that following PVV nurses’ attitudes toward violence in EDs became more negative. Nurses stated that PVV decreased job satisfaction and commitment to the work. In particularly, the withdrawal behaviors were increased in ED nurses. A previous study has highlighted the importance of caring behaviors following WPV among victims of health care providers. They showed a reduction in communications with patients and their relatives (44.7%), and changes of their post-aggression behavior at work (50.8%) [51]. As such, a patient’s specific health needs and desired health outcomes may be compromised.

PVV is highly distressing for nurses [1,3,18,52]. Nurses in this study reported their reluctant to closely contact with patients/visitors. Lanctôt and Guay [53] have also indicated that approximately 4.3–73% of victims of WPV reported fear of their patients. These negative attitudes require careful reflection regarding the influence on occupational-related outcomes such as professional nursing care behavior and quality of care. When a potential adverse consequence emerged from PVV, and high occupational risk and stress intersect, the negative effects on nurses accumulate rapidly, which is not easy to untangle and effectively manage.

Experiencing WPV is correlated with higher level of occupational stress, and largely decreased job satisfaction, job initiative, and self-efficacy. WPV is also closely linked to feeling disappointed with one’s occupation [54]. The high strain at work could linger over the following year [55]. Nurses in high work strains experienced greater exposure to interpersonal conflicts, reduced work efficiency and teamwork [56], which could trigger the occurrence of non-physical aggression during the ensuing year [55]. This is a vicious circle. Undoubtedly, WPV can be seen as an important contributing factor of health professionals’ intention to leave their occupation [54].

Handling of incidence of PVV requires an additional effort, because it includes the auspice of nurses’ professional capacity. However, nurses in our study did report loss of confidence and having a reduced self-esteem. Nurses agreed that PVV as being part of their job that they had insufficient competence to cope with these incidents. This is evidenced by the results in an earlier study that near one-third of victims of WPV had reduced confidence on the job [57]. This notion has also been demonstrated to be associated with under-reporting of violent incidents and a wrong judgment about violent situation and resources needed to mitigate WPV [1,58]. Furthermore, one of the most commonly reported consequences of WPV in any forms were frustration (one physical specific event = 45.8%, one physical ongoing event = 57.4%, one non-physical violence, combining threats, sexual harassment, and verbal abuse = 60.7%) [59]. Threats to personal integrity or pride were also reported when encountering WPV [60]. More precisely, a very high proportion of nurses (90.9%) have reported that WPV threatens their professional identity in nursing [61].

Findings from the present study particularly have shown “the fear of being retaliated against is so deep-seated”. Our data indicated that the deep fear of being retaliated against by the perpetrator if nurses reported a workplace violence incident, coupled with their negative attitudes and adverse health outcomes, have made it hard for nurses to maintain commitment to their profession. This led to anxiety, psychological distress, and lack of self-efficacy when caring of patients. Furthermore, nurses worried about the sequelae of PVV such as violence-related physical injuries occurred around the eyes. WPV caused among 85.2% of health professionals some degree of worry, and 22.1% had worried or very worried about suffering violence [62]. Thus, the judicial system, which is the most formal support system, is necessary for supporting the nurses of WPV victim. Judicial and hospital administrative support should be established and strengthened to assist nurses to obtain redress through proper procedures that are accessible. Provision of legal protection and related counseling sessions for violence victims is recommended. 

It is clear that nurses in this study recognized the gravity and complexity of PVV. Cooperation between management and front-line ED nurses is critical. Suggestions of organizational efforts and support following PVV was the last theme to explore. In Taiwan, medical WPV incidents have been repeatedly reported in the media. Frontline medical staff accept the mission of safeguarding patients’ lives and the task of managing medical care, all while facing WPV. After the high incidence of WPV was exposed by the media, this aroused criticism from many sectors of society, prompting the Legislative Yuan to rapidly pass an amendment to the Medical Law, which rendered violence in medical institutions a crime subject to public prosecution and increased the potential sentence. Continued policy is clearly warranted. The necessary renovations of security equipment to the hospital, equipments such as security cameras, emergency buttons, and other assistant facilities to secure and protect health professionals’ safety in the ED are also important and recommended.

In addition, PVV has not drawn enough attention despite its cumulative impacts of negative consequences. Therefore, it is significant to manage and prevent PVV on the organizational and individual levels in EDs. The analysis of this study indicates that a lack of knowledge and skills is a barrier for a more proactive effort of prevention of PVV. Nurses demanded practical interventions in order to increase their sensitivity to PVV before it happens. Proper training organised by the hospitals is almost recognized as an effective approach to deal with WPV, to develop knowledge and understanding of risk assessment and strategies, as well as to improve management skills tailored to specific WPV and work functioning.

No single factor could explain the etiology or the rise of PVV and the variety of perpetrator characteristics. However, nurses participated in the present study, have uncovered that alcohol abuse and mental problems are the common characteristics of perpetrators who commit violence. Extant research indicated that internal risk factors encompassed clinical and personal factors. Other studies have identified alcohol intoxication, substance abuse, and mental illness as clinical factors for PVV among perpetrators [21,22]. Regarding personal factors or behavioral problems for PVV, male and younger patients/visitors, individual propensity to anger, hostile attributions, disease and discomfort, and low socioeconomic status are all possible factors [22,25]. Environmental and process issues, and the sources of violence which constituted as external risk factors might simultaneously acted as triggers for violence in ED.

In this study, nurses also supported the need of having the workplace violence prevention training programs implemented at an organizational level. Similarly, nurses also indicated that organizational support, such as the explicit policies, plays an important part in the prevention of PVV at the workplace. Nurses in the EDs as compared to those working in other ward environments, operate in a very different clinical setting. Thus, the development strategies of management interventions for ED may need to be more specific to prevent WPV in the emergency environment. However, a better understanding of the antecedent factors and consequences of PVV is also essential for developing the interventions. Our findings regarding the needs for specific training programs are also consistent with reported in the extant studies. e.g., [63,64,65,66,67,68,69,70]. Education and training programs are key components of workplace violence prevention. A well-designed intervention could also improve nurses’ coping strategies [63,64,65].

However, by being able to stick it out through recovery times, demanding for training programs, and requesting for new knowledge, skills, and capacities of dealing with PVV, nurses have begun to form their sense of self-efficacy. Self-efficacy has been demonstrated as one of the important factors that could influence the adaptation to negative events such as occupational stressors [71]. Individuals with higher levels of self-efficacy are more officious to deal the managing workplace demands [71,72]. The positive experiences of cognitive adjustments can be developed by viewing occupational stressors as challenging situations, and making more effort to effectively managing violent events. In other words, self-efficacy beliefs have a beneficial effect specifically on nurses who experience high levels of occupational stressors, and heavy workloads related to WPV such as difficulties with patients and their visitors, difficulties in deciding how to manage the escalating violent situations, and related-physical tiredness. Therefore, self-efficacy beliefs can minimize the potential for development of adverse consequences from PVV. In addition, findings from this study have also indicated that social support from colleagues could also improve self-efficacy and lead to a better recovery following WPV [13].

This study has several limitations. Our conclusions may not be generalizable to the healthcare settings other than EDs; thus, further studies conducted in other departments or wards are needed. The present study could expand the sample size by recruiting more nurses who have left the work because of PVV. However, the merit of the present study is the sensitivity to context to which the in-depth details provided are sufficient for nurses working in the violent situation in ED. This study is specific to a PVV context, critical moments in EDs and the sample of nurses. Thus, the relatability of this qualitative study is more important than its generalizability. This study provided important information of nurses’ experiences of PVV, and their prompt and real needs in the aftermath of incidents; all of these make this study highly valuable. 

## 5. Conclusions

The present study provides insight into the dilemma, antecedents and consequences of PVV, the most common form of WPV, in ED nurses. The exploration and understanding of impacts of being threatened, intimidated or assaulted in ED nurses is significant. This paper provides compelling reasons to look beyond solely evaluating the existence of violence in the workplace, and to consider perceived professional inefficacy, impacts of being threatened or assaulted in nurses and the urgent needs and provision of prevention and management of workplace training programs to ensure the high-quality nursing care. Thus, more progress needs to be made in developing research-supported best practices and guidelines in order to mitigate and address PVV in EDs. 

## Figures and Tables

**Table 1:** Overview of themes and sub-themes.

	Main Themes	Sub-Themes		
1	Multifaceted triggers and causes of PVV	Unpredictability and complexity of diverse clinical conditions	Misestimation of violent situation	Obstacles to effective teamwork
2	Experiences following PVV	Invisible and inevitable physical and psychological trauma	Impulse to hit back	Loss of confidence
3	Tangled up in thoughts and struggle with the professional role	Encounters of feeling disrespected	Symptom sequelae following violence	Fear of retaliatory acts of violence and future violence at work
4	Self-reflexivity and adjustment	Ineffective communication skills	Stress-buffering effects of self-affirmation	Support from management and colleagues
5	Needs of organizational efforts and support following PVV	Raising awareness of violence	Guidelines and policies	Necessary renovations of security equipment

## Data Availability

The data presented in this study are available on request from the corresponding author. The data are not publicly available due to privacy and ethical restrictions.
